# Linearly Polarization-Sensitive Perovskite Photodetectors

**DOI:** 10.1007/s40820-023-01048-y

**Published:** 2023-04-07

**Authors:** Jie Sun, Liming Ding

**Affiliations:** 1 Center for Excellence in Nanoscience (CAS), Key Laboratory of Nanosystem and Hierarchical Fabrication (CAS), National Center for Nanoscience and Technology, Beijing, 100190 People’s Republic of China; 2 University of Chinese Academy of Sciences, Beijing, 100049 People’s Republic of China

## Abstract

- Polarization is an exceptional physical property of light that carries and differentiates a significant amount of optical information. Perovskite materials are utilized in polarization-sensitive photodetectors owing to their crystal structure anisotropy and controllable orientation growth, in addition to their excellent photovoltaic performance.
- This paper presents an overview of the structural characteristics and photovoltaic performance of different optical structures and low-dimensional perovskite polarization photodetectors. This summary will contribute to the future development of perovskite-based photodetectors that are sensitive to polarization.

## Graphical abstract

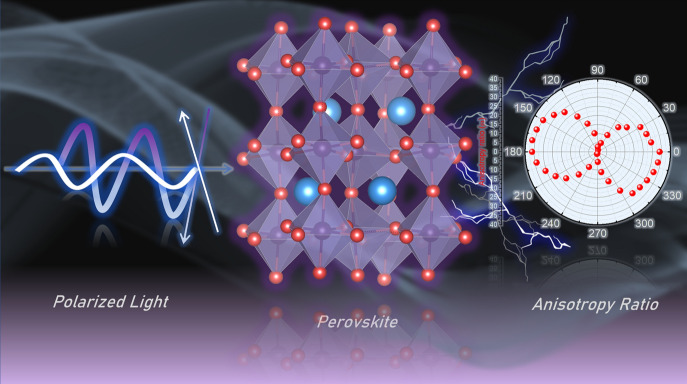

Polarization means symmetry loss for light vibration along light propagation direction, which is a particular physical property of light. The polarization state is indecipherable for most polarization-insensitive detectors. Polarization can carry and differentiate light information, and can be used in polarized light detection, polarization imaging, and encryption communication [1]. Photodetectors are the core component of some photoelectric devices [2] used in biomedical sensing, remote sensing, military field, etc.

An excellent photodetector (PD) can recognize all the properties of light, including intensity, frequency, and polarization. PDs capable of detecting polarized light need a specific optical structure or crystal structure. Lately, perovskite was used in polarization-sensitive PDs due to its controllable orientation growth and crystal structure anisotropy. In addition, compared with other polarization-sensitive materials like graphene [3], metal halide perovskites possess excellent photovoltaic performance. The strong light absorption and high carrier mobility of perovskite can be combined with its ability to recognize polarized light, thus yielding self-powered polarization-sensitive perovskite photodetectors (P-PPDs).

CsPbX_3_ perovskites were found to emit polarized light both in solution and in films in 2016 [4]. The study inspired people to explore the polarization detection capability of perovskites when used in optoelectronic devices. The research of P-PPDs was divided to linearly polarized light (LPL) and circularly polarized light (CPL). The electric field vector of CPL is rotary, so CPL has an axisymmetrically uniform distribution of the scattered field. Unlike CPL, the polarization direction of LPL is fixed, so linearly polarization-sensitive perovskite photodetectors (LP-PPDs) can detect periodic photoelectric signal changes. LP-PPDs can be realized by constructing an optical structure or by controlling crystal structure, which profits from the controllable growth orientation of perovskite and the anisotropy of lattice structure, respectively.

The optical structures of LP-PPDs refer to patterned perovskite active layers, including nanowire (NW) arrays, nanoribbon (NR) arrays, and so on. Most of these structures were fabricated by nano-imprinting, etching, or one-step self-assembly. Tang et al. [5] made LP-PPDs by one-step self-assembly of single-crystalline CH_3_NH_3_PbI_3_ NW arrays. The large length/width ratio of these 1D nanowires led to an anisotropy of 1.3. In addition, they improved the stability of CH_3_NH_3_PbI_3_ NWs by using oleic acid to passivate the surface defect of perovskite, obtaining a detectivity of 2 × 10^13^ Jones. Jiang et al. [6] prepared a 1D CsPbBr_3_ single crystal with rigid crystallographic alignment through an effective solution-processing method and assembled it to make LP-PPDs. The device realized an anisotropy ratio of 2.6, a dark current of 8.13 × 10^–10^ A, and a light on/off ratio of nearly 10^3^. Ko et al. [7] used spin-coating method with solvent treatment to fabricate CH_3_NH_3_PbI_3_ NR arrays and LP-PPDs. Compared with CH_3_NH_3_PbI_3_ thin-film PPDs, NR arrays-based LP-PPDs showed higher detectivity due to effective photon management of grating-like NR structure. In the same year, Tang et al. [8] demonstrated a β-CsPbI_3_ NW-based LP-PPDs with a high anisotropy ratio of 2.68, which is also suitable for flexible substrate. The flexible device exhibited an anisotropy ratio of 2.17 and a low loss of photoelectric performance after 500 bending cycles. Though the above patterned structure-based LP-PPDs increased the polarization dimension of light, reducing the optical loss is crucial. Li et al. [9] designed a G-PC-PD by bonding a 1D nanograting with porous 2D photonic crystal (PC), which was inspired by the hierarchical architecture of the butterfly. The combination of 2D PC and nanograting contributed to the excellent light-harvesting ability of G-PC-PD, showing more than six times higher responsivity and detectivity than that of flat-film perovskite photodetectors. In 2021, a moiré LP-PPD with a double-nested grating was reported by Li et al. [10] Taking advantage of the waveguide effect of double-nested grating, and enhanced light-harvesting ability of top and bottom grating, a high responsivity of 15.62 A W^−1^ and a detectivity of 5.58 × 10^13^ Jones were achieved, respectively. The different optical structures of CH_3_NH_3_PbI_3_ are shown in Fig. 1. There are many other perovskite materials like CH_3_NH_3_PbBr_3_ and CH(NH_2_)_2_PbI_3_ served as surface-patterned LP-PPDs [, 11, 12] (Fig. 2). Though surface artificial nanostructure assists optical management and polarization of PDs, the perovskite instability is inescapable. Table 1 summarizes LP-PPDs based on different optical structures. Recently, Zhang et al. [13] reported PDs with in-situ encapsulated moiré lattice, which consist of two soft templates of nano-grating with rotation angles. The moiré lattice of CH_3_NH_3_PbBr_3_ led to strong light-harvesting capability and high anisotropy. The moiré LP-PPDs showed an ultrahigh detectivity of 1.05 × 10^14^ Jones, a responsivity of 1026.5 A W^−1^, and an anisotropy ratio of 9.1.

**Fig. 1:**
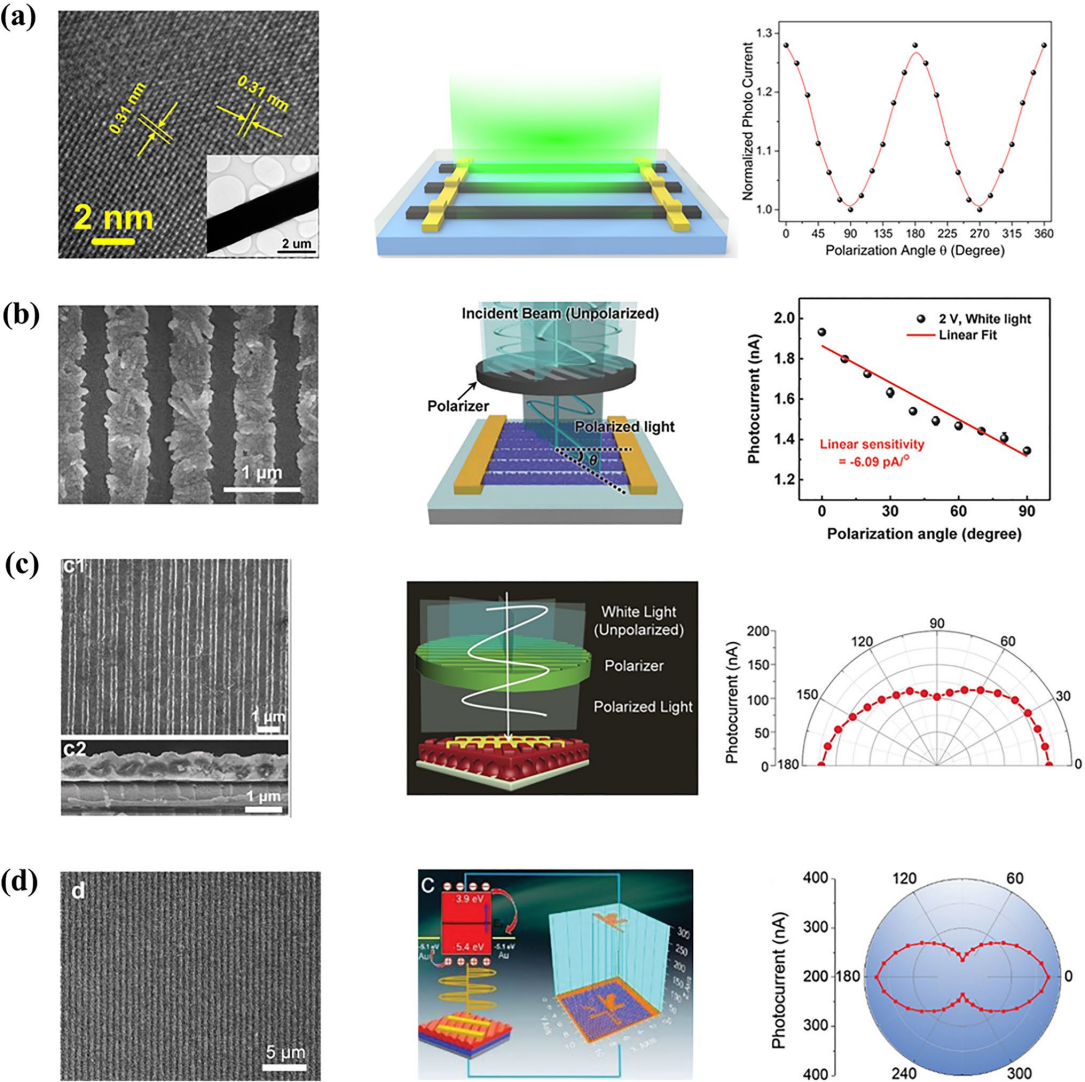
Performance of LP-PPDs based on CH_3_NH_3_PbI_3_ with different optical structures. **a** 1D nanowire arrays. Reproduced with permission [5], Copyright 2016, American Chemical Society. **b** 1D nanoribbon arrays. Reproduced with permission [7], Copyright 2018, John Wiley and Sons. **c** 1D nanograting with 2D photonic crystal. Reproduced with permission [9], Copyright 2019, John Wiley and Sons. **d** Stacked dual grating. Reproduced with permission [10], Copyright 2021, John Wiley and Sons

**Fig. 2:**
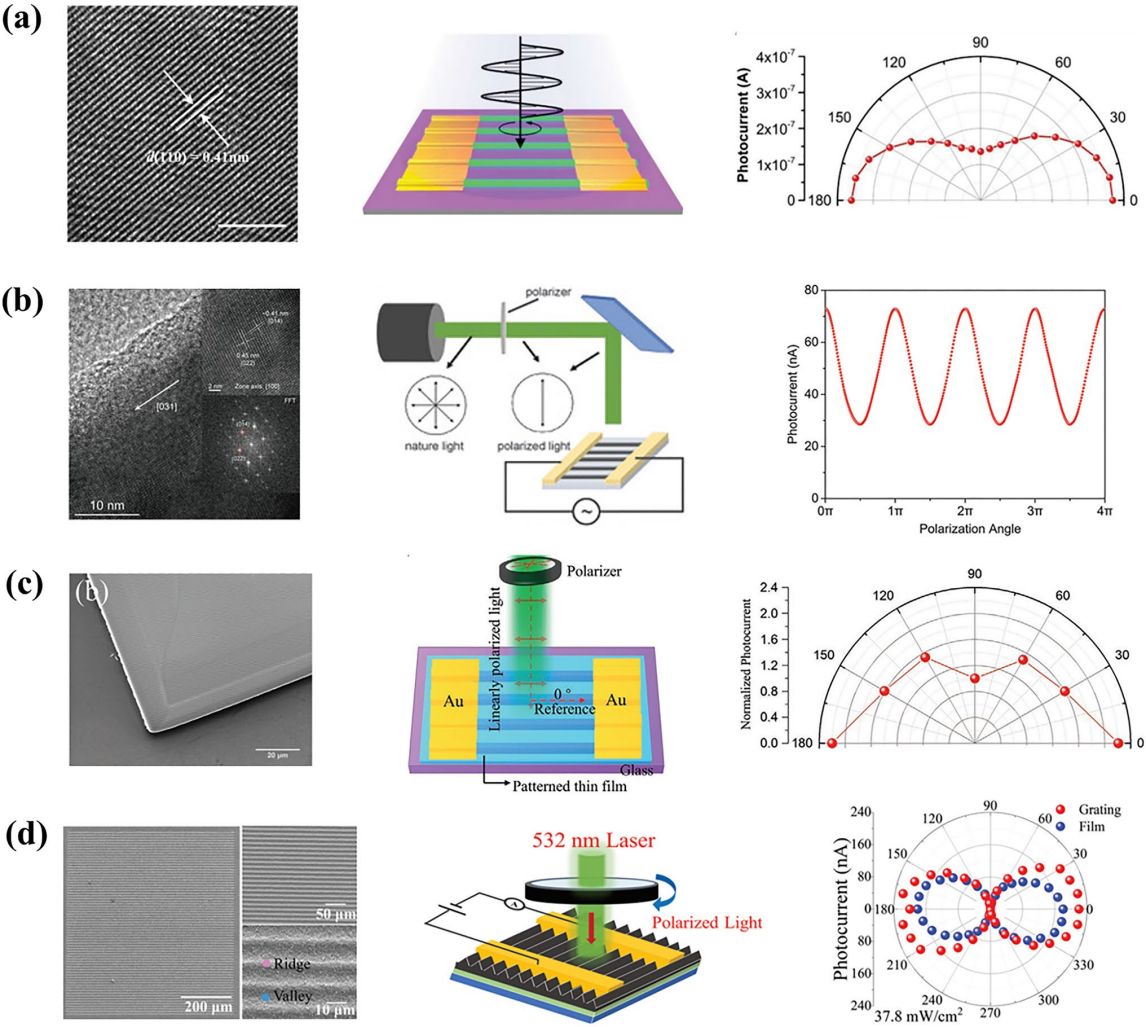
Performance of LP-PPDs based on different perovskites. **a** CsPbBr_3_. Reproduced with permission [6], Copyright 2017, John Wiley and Sons. **b** β-CsPbI_3_. Reproduced with permission [8], Copyright 2018, John Wiley and Sons. **c** CH_3_NH_3_PbBr_3_. Reproduced with permission [11], Copyright 2021, John Wiley and Sons. **d** CH(NH_2_)_2_PbI_3_. Reproduced with permission [12], Copyright 2022, John Wiley and Sons

**Table 1:** Performance for optical structure-based LP-PPDs

Active Layer	Optical Structure	Wavelength (nm)	Anisotropy Ratio	Detectivity (Jones)	Responsivity (A W^−1^)	Response Time (s)	On/Off Ratio	References
CH_3_NH_3_PbI_3_	1D nanowire array	530	~ 1.3	2 × 10^13^	4.95	< 10^–3^		[5]
CsPbBr_3_	1D nanoribbon array	470	2.6		1.4 × 10^3^	2.15/2.34 × 10^–5^	< 10^3^	[6]
CH_3_NH_3_PbI_3_	1D nanoribbon array	300–800		1.76 × 10^11^	2.2 × 10^–3^	2.72/2.62 × 10^–2^		[7]
β-CsPbI_3_	1D nanowire array	530	2.68	3.46 × 10^10^	7.45 × 10^–1^			[8]
CH_3_NH_3_PbI_3_	1D nanograting with 2D photonic crystal	620/620/750	1.6	3.22 × 10^13^	12.67	2.1/6.7 × 10^–2^	5.87 × 10^3^	[9]
CH_3_NH_3_PbI_3_	Stacked dual grating	650	1.58	5.58 × 10^13^	15.62	1.12/0.63 × 10^–3^	2.70 × 10^4^	[10]
CH_3_NH_3_PbBr_3_	Single crystal nanograting	532	2.2	1.08 × 10^10^	8 × 10^–3^	0.1		[11]
CH(NH_2_)_2_PbI_3_	Grating structure	532		7.8 × 10^12^	11.7		1.01 × 10^3^	[12]
CH_3_NH_3_PbBr_3_	Two identical nanograting structure	650	9.1	1.05 × 10^14^	1026.5	3.0/2.3 × 10^–3^		[13]

The structure for low-dimensional perovskites exhibits completely different optoelectronic properties from that of 3D perovskites. The optical anisotropy might be due to different bonding characteristic [14]. Using macromolecules to separate 3D perovskite is an effective way to realize polarization-sensitive detection. In 2019, 2D perovskite (*iso*-BA)_2_PbI_4_ single crystals were prepared to make a narrowband LP-PPD [15]. (*iso*-BA)_2_PbI_4_ possesses enhanced anisotropy, yielding a detectivity of 1.23 × 10^10^ Jones and an anisotropy ratio of 1.56 (Fig. 3a). Li et al. [16] also designed 2D perovskite [CH(NH_2_)_2_][C(NH_2_)_3_]PbI_4_ (FAGPbI_4_) with corrugated inorganic layer. The high anisotropy of FAGPbI_4_ was attributed to the existence of [PbI_6_]^4−^ layer, offering an anisotropy ratio of 2. The polarized light can be produced by some crystal planes through the regulation of temperature. Although hybrid organic–inorganic 2D perovskite came to be used in polarization-sensitive photodetection, the synthetic method for high-quality 2D perovskites is still being explored. Sun et al. prepared 2D perovskite (FPEA)_2_PbI_4_ with low trap density by a minute-scale rapid crystallization [17]. And the high anisotropy ratio (2.1) of LP-PPD was thought to be caused by the physical property of 2D quantum-well structure, composed of organic cation barriers and inorganic perovskite wells (Fig. 3b). Besides, 2D inorganic perovskite advances in polarization-sensitive photodetection. In 2020, TRA was used to prepare 2D perovskite, yielding an anisotropy ratio of 2.1, and an on/off current ratio over 10^4^ [18]. Though the crystal structure of 3D CsPbBr_3_ is isotropy, some molecules' introduction can turn it into anisotropy. Sun et al. also synthesized a Dion-Jacobson (DJ) type 2D perovskite (HDA)CsPb_2_Br_7_ by alloying diammonium into 3D CsPbBr_3_ [19]. The device exhibited an anisotropy ratio of 1.6, a detectivity of 1.5 × 10^9^ Jones, and a high phase stability in environmental conditions. Some other 2D perovskites can improve the performances of LP-PPDs, and the anisotropy ratio reached 6.8 [20] (Fig. 3c), the detectivity and on/off ratio reached 1.53 × 10^12^ Jones and 3 × 10^8^ [21] (Fig. 3d), respectively. Table 2 summarizes the performance of low-dimensional LP-PPDs.

**Fig. 3:**
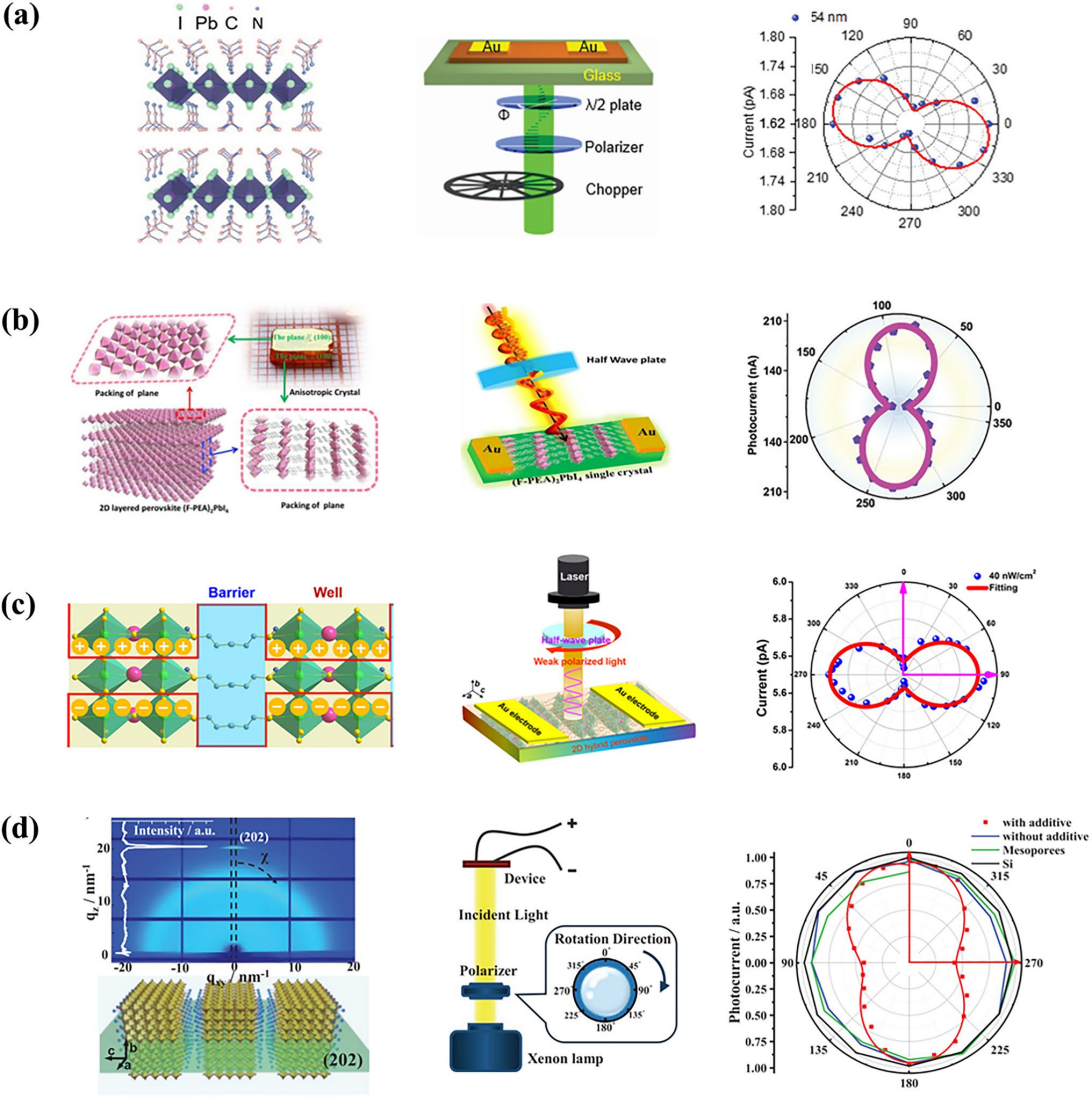
Structure of different 2D perovskites, and the performance of LP-PPDs with different 2D perovskites. **a** (*iso*-BA)_2_PbI_4_. Reproduced with permission [15], Copyright 2019, John Wiley and Sons. **b** (FPEA)_2_PbI_4_. Reproduced with permission [17], Copyright 2020, John Wiley and Sons. **c** (BPA)_2_PbBr_4_. Reproduced with permission [20], Copyright 2021, Elsevier. **d** PEA_2_MA_4_(Sn_0.5_Pb_0.5_)_5_I_16_. Reproduced with permission [21], Copyright 2021, John Wiley and Sons

**Table 2:** Performance for low-dimensional LP-PPDs

Active Layer	Wavelength (nm)	Anisotropy Ratio	Detectivity (Jones)	Responsivity (A W^−1^)	Response Time (s)	On/Off Ratio	Refs.
(*iso*-BA)_2_PbI_4_	552/560	1.56	1.23 × 10^10^	0.56	2.33/1.66 × 10^–1^	10^3^	[15]
[CH(NH_2_)_2_][C(NH_2_)_3_]PbI_4_	515	2	2 × 10^10^				[16]
(FPEA)_2_PbI_4_	520	2.1	7.45 × 10^11^	3.2	4.3/4.6 × 10^–4^		[17]
(TRA)_2_CsPb_2_Br_7_	405	2.1	7.45 × 10^10^	1.42 × 10^–3^	3.2/3.8 × 10^–1^	1.3 × 10^4^	[18]
(HDA)CsPb_2_Br_7_	405	1.6	1.5 × 10^9^	2.1 × 10^–4^	2/3 × 10^–4^		[19]
BA_2_CsPb_2_Br_7_	405	1.5	1.2 × 10^12^	3.95 × 10^–2^	3 × 10^–4^	4.6 × 10^3^	[22]
(BPA)_2_PbBr_4_	377	6.8	10^7^	10^–4^	2.7/3.0 × 10^–2^	10^4^	[20]
PEA_2_MA_4_(Sn_0.5_Pb_0.5_)_5_I_16_	520/625/900	2.4	1.53 × 10^12^			3 × 10^8^	[21]
(BA)_2_(GA)Pb_2_I_7_	520	2.2	3.3 × 10^11^	1.2 × 10^–2^		2 × 10^3^	[23]

In short, only specific perovskites can detect light polarization, based on the anisotropic crystal structure or the anisotropy of optical structure. Linearly polarization-sensitive perovskite photodetectors have been advancing since 2016. More efforts are needed to explore the method for achieving high anisotropy and stability.
